# Reconceptualizing Workplace Thriving and Setting Future Research Agenda

**DOI:** 10.5334/pb.1416

**Published:** 2026-02-19

**Authors:** M. M. Sulphey

**Affiliations:** 1 College of Business Administration, Prince Sattam Bin Abdulaziz University, AlKharj, Saudi Arabia

**Keywords:** Reconceptualisation, thriving, workplace thriving, WPT

## Abstract

Workplace Thriving empowers individuals to thrive in their jobs and flourish in their careers. It is conceptualized as a psychological state in which individuals experience vitality and learning. Limiting thriving to these two has reduced its applicability despite the concept being interconnected, multi-dimensional, and holistic. This work examines thriving, identifies the shortcomings of the existing literature, and proposes a fresh perspective for reconceptualizing it. After defining the construct as “a state of positive engagement and well-being derived when employees are mindful, resourceful, and resilient, enabling them to navigate organisational challenges and achieve effectiveness,” the study proposes a fecund conceptual model. The study focuses on contextual, proximal, and resource factors, such as deriving mindfulness, resourcefulness, and resilience. This reconceptualization presents arguments for understanding thriving, enabling individuals to navigate their organizational lives more effectively.

## Introduction

In the fast-paced world, the success of any organization depends on creating a workplace environment of sustainability, where employees can thrive. Recent Gallup (2025) findings show that globally, only 21% of employees are engaged, 33% thrive, and the majority feel emotionally drained at work. This is because the dynamic, volatile work environment increases stress, ambiguity, and cognitive overload, leading to higher burnout and disengagement. Thriving is not merely the absence of strain but the presence of growth-oriented energy that enables employees to adapt, innovate, and perform sustainably. Workplace thriving (WPT) is grounded in the inherent drive of human beings toward self-improvement and growth (Maslow, 1965; Ryan & Deci, 2002). Research interest in WPT is now burgeoning due to its perceived ability to develop people, deliver happiness, foster a sense of accomplishment, and support and reward supportive and rewarding relationships in their careers (Spreitzer and Porath, 2014; Su et al., 2014; Usman et al., 2022). Thriving is a complex concept with varying connotations and conceptualizations across individuals, domains, and disciplines, making it challenging to establish a universal definition. Contextual variability has also led scholars and researchers to adopt multiple conceptualisations depending on the domain and discipline studied (Brown et al., 2017). The Cambridge Dictionary identifies thriving as “prosperous and growing” and “flourishing”. It helps individuals by working as an internal gauge in understanding their psychological development (Spreitzer and Porath, 2014).

Workplace Thriving (WPT) was initially defined in medical terms by Spreitzer et al. (2012), who proposed that failure to thrive involves an inability to grow, which can manifest as a lack of appetite and stunted growth. In the Organisational behavior context, WPT refers to a psychological state in which an individual experiences a sense of vitality and learning, shaped by contextual factors (Spreitzer et al., 2005). The literature on WPT has multiplied since the work of Spreitzer et al. (2005). According to Zhao et al. (2025), WPT involves a psychological state characterized by energy and continuous acquisition of knowledge and skills in the rapidly changing current work environment. WPT facilitates employees’ success in their positions within their organisations and helps them flourish through personal development, which can lead to career success (Wallace et al., 2013). According to Yang et al. (2019), WPT indicates achieving optimal activities in terms of “goodness, generativity, growth, resilience, or a personal state to perform mental and social duties.” It produces all-around “contagious” favourable outcomes for all stakeholders (Porath et al., 2012; Um-e-Rubbab et al., 2021). Thriving employees are capable of making behavioural adjustments that help them fit into situations and be effective (Spreitzer et al., 2005) and become psychologically engaged while executing higher-order duties (Spreitzer and Porath, 2014). Thus, WPT encapsulates employees’ psychological development at work. (Porath et al., 2012). It is a scale that helps individuals psychologically understand their subjective growth, and is distinct from employee well-being and self-actualization (Porath et al., 2012; Spreitzer & Porath, 2014). Thriving people feel progress and momentum. This paper aims to introduce and explore WPT as a derivative of the overarching concept of thriving. It is dynamic, constantly evolving, interacting, and influenced by multiple factors. According to Coe-Nesbitt et al. (2021), thriving is an interconnected, multi-dimensional, and holistic concept. Haight et al. (2002) identified three interacting factors in the thriving continuum—the person, the human, and the nonhuman environment —which continuously interact and change. Elements of the human environment, including the various individuals encountered throughout life, can shape the surrounding environment and, in turn, support or hinder thriving. Jiang et al. (2023) identified positive affect resources, particularly highly activated positive affect, as playing a significant role in WPT.

The study will help to better understand WPT’s microfoundations and dynamics by discussing its diverse perspectives and counterarguments, providing an equitable assessment. Recognising the potential of WPT, the study also examines the construct’s significance and its applicability in the contemporary, complex, and dynamic work environment. A review of the research reveals multiple distinct conceptualisations of WPT, each reflecting diverse implicit assumptions about the concept. This work examines the current conceptualisation of WPT, identifies shortcomings in the existing literature, and proposes a fresh perspective for reconceptualising WPT. The study also develops a conceptual model of WPT. The study’s findings could also inform better future directions for examining this exciting construct and further developing it.

Many concepts in the literature are mismatched or misaligned. Researchers seek to explore and explain such misalignments based on their observations of the concept, as they could create “analytical and theoretical blind spots” (Knott & Alejandro, 2024, p. 1). Such blind spots tend to foreclose the prospects of further probing and articulating the observed conceptual specificities and complexities. This opens up multiple vistas for reconceptualising existing concepts. This approach will present researchers and social scientists with a more nuanced and better-adapted concept, having better analytical, theoretical, and empirical leverage. There is a large body of literature on WPT, and the present work does not intend to analyze it in detail. This study aims to demonstrate that reconceptualisation can shed further light on the reasons behind the multiple understandings and definitions of WPT. This work reconceptualizes WPT by addressing the gap in the literature and presenting a conceptually strong solution to this misalignment. During the process of reconceptualisation, several reasoning methods are used. While the study employed deductive reasoning to gain a deeper understanding of the concept, inductive reasoning helped establish a robust basis for reconceptualisation.

## Methods

This paper aims to advance the understanding, theorising, and reconceptualisation of WPT. In addition, this study examines its nature, core factors, and dynamics. The study utilized “theoretical elaboration” to achieve this objective. This elaboration is based on a focal point, posing that any concept, theory, or research domain is inherently imperfect (Jaakkola, 2020). Theoretical elaboration involves theorising and empirical research based on conceptual frameworks or an initial model. This process generates novel theoretical insights by refining, contrasting, or organising theoretical constructs and relationships to justify and elucidate empirical findings (Fisher & Aguinis, 2017). This study used several techniques for theory elaboration, as proposed by Fisher & Aguinis (2017). They included horizontal and vertical contrasting, specification of the constructs, splitting the prevailing constructs, and structuring the various relationships. This is achieved by connecting with empirical data through an iterative process of theorization, as proposed by Lindgreen et al. (2021). This study identifies the dynamics of WPT to address shortcomings in the existing literature and to develop a conceptual process model.

This work is based on the premise that WPT lacks a universal definition and that no single meaning exists. This is usually true for any concept in social studies. The following issues must be addressed to reconceptualise any construct and add value (Sinkovics et al., 2015). There is a definite need to propose a precise definition of the construct. The reconceptualisation must refine earlier conceptualisations, taking their complexity into account and presenting their advantages and limitations. This study adopts a pluralist approach to reconceptualisation, drawing on the linguistic method advocated by Berenskoetter (2017), Guzzini (2013), and Ish-Shalom (2021), as well as the “ladder of abstraction” proposed by Sartori (1970). The linguistic approach involves reflexive attention to concepts, the presentation of accurate and accessible solutions, and the resolution of scholarly disagreements over language (Knott and Alejandro, 2024).

This study aims to foster a constructive discourse on WBT and to reconsider its boundaries. The paper is presented as follows. The following section provides a general overview of existing conceptualisations, followed by a detailed description of the reconceptualisation. The first section elaborates on prior work on WPT, identifying gaps, conflicts, and underexplored areas in both the theoretical and empirical domains of the concept. This section follows an evaluation of current WPT definitions. After that, an attempt is made to redefine WPT, which also involves refining or creating new definitions. A framework is then developed to organise the construct, presenting the expected interactions and consequences. This section will also decipher the concept in terms of specific metrics that can be empirically tested, providing a valid basis for testing, validation, and adaptation to various research scenarios. Hence, this framework will translate abstract theoretical concepts into specific operational metrics that can be empirically examined, thereby enhancing its utility across studies and contexts. Thus, based on a structured approach, this paper presents a cohesive framework for understanding WPT, establishes a robust theoretical foundation, and advances empirical research. The focus on WPT is indispensable for any organization, as it involves individual growth and momentum achieved through feeling energized, flourishing, and continually improving at work.

## Literature review

Research about thriving has its origin in the works of Maslow (1943, 1954), Rogers (1961), and Alderfer (1972). According to Maslow (1954), self-actualization represents the positive extreme of thriving, characterized by peak experiences and optimal performance, with individuals living life to the fullest. Thriving depends on interactions with dynamic environmental factors and ongoing self-development. It is fluid, as all factors that influence it continually change and interact, resulting in a gestalt. These factors can impact both the environment and the individual, contributing to optimal growth, development, and thriving, or even hindering it. A workplace that thrives depends on individual work contexts (Spreitzer et al., 2005). It is strongest in workplace environments that promote discretion in decision-making, significant knowledge sharing, or an atmosphere of mutual respect and trust. Contextual features encourage individuals to work agentically (by meeting basic employee psychological needs), resulting in a better sense of knowledge and ownership, favourable meaning, affective and relational resources (Spreitzer et al., 2005), and psychological attachment (Usman et al., 2022; Weng et al., 2010).

### Defining WPT

Thriving, a positive construct that exists as a continuum, is defined differently. In the psychology literature, flourishing is defined as a dynamic process of adapting to physical, psychological, or social adversity, resulting in favourable outcomes involving individual growth and improved conditions (Bugental, 2004; Jackson et al., 2007). According to Haight et al. (2002), thriving is “the ongoing process of growing through continuous human-environment interactions, resulting in social, physical, and psychological resilience and growth.” Schreiner (2010, p. 4) states that thriving occurs when individuals are “fully engaged intellectually, socially, and emotionally.” It is also “a desirable life condition” (Bundick et al., 2010) and can foster positive effects (Carver, 1998; O’Leary & Ickovics, 1995). It is the ability to bounce forward from stressful events, reaching a higher level of health or functioning (Haas, 2015; Smith et al., 2008). Haight et al. (2002) highlighted the importance of resilience when they defined thriving “as the ongoing process of growing through continuous human-environment interactions, resulting in social, physical, and psychological resilience and growth.” Spreitzer et al. (2005) proposed that individuals are more likely to thrive when specific enabling conditions are present in the workplace, while minimizing constraints.

According to Spreitzer et al. (2005), thriving is a second-order composite construct that jointly has vitality and learning at work. Vitality refers to the positive emotions and energy required to maintain enthusiasm (Nix et al., 1999). Learning encompasses the acquisition and application of knowledge and skills to enhance work competence and confidence (Carver, 1998). Vitality is the positive energy that effectively drives work. A thriving individual strives for a better life, which can be achieved with learning (Spreitzer et al., 2019). Spreitzer et al. (2005) state that the absence of either dimension will lead to limited WPT. By monitoring thriving levels, employees’ behaviors are fine-tuned to be effective (Spreitzer et al., 2005). The same opinion was expressed by Porath et al. (2012). They identify WPT as involving growth and momentum, characterized by feeling energized and alive and continually improving at work. While the former is known as vitality, the latter is known as learning. WPT involves employees’ psychological growth and advancement in the workplace. It differs from employee well-being, self-actualization, and other specific characteristics (Chang and Busser, 2019; Porath et al., 2012). Thus, during thriving, there are upward individual trajectories (Hall et al., 2009; Joseph & Linley, 2008; Thomas & Hall, 2008; Zhao et al., 2025).

A review of available definitions primarily highlights the multifaceted nature of thriving (Brassey et al., 2024; Spreitzer et al., 2005; Brown et al., 2017) and its self-sustaining mechanism (Ellaban et al., 2025; Goh et al., 2022). The literature highlights two recurring joint themes – development and triumph. While the former is associated with progressive enhancements that could be physical, psychological, or social, the latter denotes various temporally and contextually relevant outcomes. The two components work in tandem rather than in isolation. According to Su et al. (2014), achieving these two goals requires individuals to practice holistic functioning. Well-being refers to the state of succeeding in life and encompasses dimensions such as social (Haight et al., 2002; Keyes, 1998), physical (Scheier & Carver, 1987), emotional (Keyes, 2002), and psychological (Ryff, 1989). Performance is the quality displayed in executing an operation, process, or accomplishment (Simpson et al., 1989). It is measured across various motor, cognitive, or other work-related tasks. Task performance reflects an individual’s level of functioning, with superior performance leading to success (Brown et al., 2017; Sarkar & Fletcher, 2014). Haight et al. (2002) suggest that thriving can lead to social, physical, and psychological resilience and growth. It can be observed that the various definitions of WPT fail to converge, offering an array of domains and sub-variables. The key domains that emerged from the multiple definitions reviewed include vitality, learning, holistic functioning, engagement, mindfulness, and resilience.

### Theoretical underpinnings

Multiple theories have been found to advance the concept of thriving (Paterson et al., 2014; Spreitzer and Porath, 2014; Usman et al., 2023). A few such theories are discussed in the following sections:

The theory of self-adaptation (Tsui and Ashford 1994) offers insights about thriving. Self-adaptation entails the process by which humans direct goal-oriented activities over time in response to changing circumstances. Self-adaptation regards individuals as rational and independent entities, focusing on goal formulation, self-monitoring, self-reward, and self-punishment, which they employ to manage their actions (Porath and Bateman, 2006). This view overlooks the idea that individuals can control their emotions. Thriving is a valuable subjective experience that empowers individuals to examine if their approaches and activities foster positive growth. Positive growth involves progress in the short term and adaptability to the work environment in the long term (Kolb, 1984). Individuals use thriving as a metric to determine the necessity and manner of their actions in the workplace to sustain or restore it.

The model developed by Spreitzer et al. (2005) describes thriving as a “desirable informative” state. It is ‘desirable’ because individuals are motivated to enhance their thriving, and ‘informative’ since it indicates whether they are on a positive developmental trajectory. They define flourishing as a “subjective meter” determining the direction of their professional progress. Thriving encourages people to pursue behaviors that facilitate progress, thereby enabling effective navigation of the work environment. This model links WPT with agentic behaviors.

Self-determination theory focuses on the social and contextual conditions that influence the natural processes of intrinsic motivation and healthy psychological development (Ryan & Deci, 2020). It examines the factors that could enhance or undermine motivation, self-regulation, and well-being. The theory postulates that basic psychological needs — autonomy, competence, and relatedness — are essential components without which thriving cannot occur. These components lead to agentic work, which refers to actions driven by individual initiative, ingenuity, autonomy, and self-direction, where individuals act independently. Agentic work also enables thriving through personal responsibility, proactive behaviors, and the ability to influence one’s work environments. Spreitzer and Porath (2014) used this theory to explain the relationship between work behaviors and thriving.

Another applicable theory is the Conservation of Resources (COR) theory. The theory posits that individual behaviors are influenced by the gain or loss of their resources (Hobfoll, 1989), as they voluntarily invest their available resources to gain additional resources. Based on COR, Usman et al. (2022) propose that individuals strive to conserve resources to link WPT and knowledge hiding. They opined that thriving would reduce knowledge hiding, as individuals with WPT will likely use the knowledge resource to trade off, expecting additional personal gains. Thus, WPT exerts its influence on discouraging individuals from withholding knowledge.

The socially embedded model is another central framework for studying WPT (Spreitzer et al., 2005). This model suggests that WPT is shaped by contextual factors, and resources generated through agentic work behaviors are controlled by exploration, task focus, and mindful interactions (Bandura, 2001). It also involves the social conditions that either enhance or reduce the positive features that promote intrinsic motivation. According to the model, thriving is a subjective indicator distinguishing whether positive development occurs (Spreitzer et al., 2005). Empirical evidence also supports the socially embedded model, as evidenced by the positive relationship between thriving and task focus, mindful relating, positivity, and exploration (Niessen et al., 2012; Paterson et al., 2014; Usman et al., 2022). Jiang (2017) observed that WPT reflects a proactive personality. Further, favorable contextual and personal aspects enhance WPT, reducing turnover intentions (Chang and Busser, 2020).

### Distinguishing from other terms

Specific terms may appear similar to thriving, but they differ in fundamental ways. A few such terms include “prospering” and “resilience,” among others. Prospering involves the success component of thriving (Soanes & Stevenson, 2005) but not the developmental facet. Resilience is another term associated with thriving. It reflects a positive ability to adapt to adversity. Resilience enables continued functioning after an adverse event (Bonanno, 2004). However, thriving involves establishing enhanced functioning (O’Leary and Ickovics, 1995) and is not dependent on adverse events (Brown et al., 2017; Sarkar and Fletcher, 2014). It can occur following adversity and opportunity (Feeney and Collins, 2015). It can help individuals achieve optimal well-being, progress, resilience, and a sense of personal well-being in all their activities, enabling them to fulfill their mental and social responsibilities.

Spreitzer et al. (2019) related thriving to concepts such as flow, flourishing, subjective well-being, self-actualization, and resilience, while conceptualizing that WPT is distinct from these concepts. Flow involves a holistic sense of an individual absorbed in their activities. It is a state of holistic absorption in which an individual is fully engaged in their activities. In this state, individuals experience a deep sense of engagement and active participation (Piniel and Albert, 2019). Flourishing involves displaying positive emotions and functioning effectively in life, which are considered signs of mental health (Keyes, 2003). The two constructs differ, although they involve individual experiences of development and success (Benson and Scales, 2009; Spreitzer et al., 2005). Although both encompass subjective well-being (Keyes, 2007; Keyes & Waterman, 2003), flourishing involves both psychosocial and emotional well-being (Fredrickson, 2006). However, thriving is distinct in that it consists of both well-being (mental and physical) and performance components (Sarkar & Fletcher, 2014; Sheldon et al., 2001). WPT involves achieving optimal positive experiences — such as goodness, growth, and resilience — associated with human development, which manifest as vitality and learning. Thus, despite this apparent similarity, these are distinct constructs.

Several empirical studies have identified the antecedents and consequences of WPT, which are discussed in the following section.

### Antecedents and consequences of WPT

Since thriving is multifaceted, a mere subjective perception of higher levels on a single index would not be ideal for achieving development and success (Brown et al., 2017). Hence, an individual who perceives high performance and experiences low well-being may still succeed. However, this could be accompanied by adverse outcomes, including impaired development and burnout (Spreitzer et al., 2005). On the contrary, if an individual experiences high well-being but perceives low performance, task execution may be impaired, hindering success. Thus, thriving is a unique state that responds to situations.

Social scientists have examined the outcomes derived from thriving. WPT produces “contagious” positive outcomes for employees, organizations, and other stakeholders (Porath et al., 2012). Cao et al. (2023) and Ryan and Frederick (1997) found vitality related to self-determination, well-being, self-actualization, and performance. Ellaban et al. (2025) and Peters et al. (2021) state that thriving individuals are less likely to have chronic health conditions. Some other outcomes include creativity and innovation, engagement, and career adaptability (Collins, 2014; Elsayed et al., 2025; Prem et al., 2017; Um-e-Rubbab et al., 2021). According to Jiang (2017), those with higher levels of WPT transmit proactive personality and career adaptability. Carmeli and Spreitzer (2009) found that thriving is related to innovative behaviour. Benson and Scales (2009) observed that thriving results in explicit spiritual development and prosocial orientations. Kleine et al. (2019) identified attitudinal, health, and performance-related outcomes of WPT. While attitudinal outcomes include commitment and reduced turnover intentions, health outcomes include subjective health and reduced burnout. A few performance-related outcomes include organizational citizenship behavior, creativity, and team performance.

Thus, thriving employees do not suffer burnout (Hall, 1998). Thriving also reduces job strain and increases general health and well-being. Since promoting thriving is cost-efficient as it reduces absenteeism and stress (Leiter & Maslach, 2005), managers must promote it (Rai et al., 2024; Porath et al., 2012). Further, thriving fosters engagement, and engaged individuals are energetic and strive to achieve organizational goals (Harter et al., 2002). Spreitzer et al. (2005) state that thriving is an adaptive developmental process. Hence, low thriving automatically triggers self-regulation adjustments. Chang and Busser (2020), Jiang et al. (2017), and Porath et al. (2012) identified WPT as socially embedded and associated it with career adaptability and retention. Thus, WPT is a potent force that ignites sparks and energizes, driving healthy, high-performing employees and fostering human sustainability (Ding and Chu, 2020). A lack of thriving will reduce an individual’s appetite to grow and impair personal and professional development (Ding and Chu, 2020; Umm-e-Rubbab et al., 2021).

### Operationalization

An in-depth literature review revealed that WPT was primarily operationalized using the two-factor measure developed by Porath et al. (2012). The factors are Vitality (“a psychological state marked by enthusiasm and spirit”) and Learning (“the sense that one can acquire and apply valuable knowledge and skills”). Their measure consisted of five items derived from Ryan and Frederick’s (1997) subjective vitality scale and five newly developed items for a “momentary sense of learning at work” (Porath et al., 2012, p. 256). Atwater and Carmeli (2009) also developed and validated similar operationalizations. Rozkwitalska (2016) and Basinska (2017), in line with previous operationalizations, examined the learning dimension using the “Learning goal orientation scale” developed by Vandewalle (1997).

Though adequate literature on WPT has accumulated over the past few years, it remains scattered (Table 1). A lack of comprehensive knowledge of the nomological network of WPT prevents the development of specific, consistent conceptualizations and recommendations for future research and organizational practice. This calls for creating common ground through conceptual dialogue regarding the philosophically and empirically identified assumptions, as Eigenbrode et al. (2007) proposed.

**Table 1:** Operationalization of WPT.


	AUTHORS	FACTORS	NUMBER OF ITEMS

**1**	Porath et al. (2012)	Vitality Learning	10

**2**	Na-Nan et al. (2020)	Vitality Learning	12

**3**	Peters et al. (2021)	Psychological, Emotional, Social, Work-life integration, Basic needs, Experience of work, Health.	87

**4**	Smith et al. (2023).	A single factor of thriving	6

**5**	Neidlinger et al. (2024)	Work-related emotional & psychological well-being Social well-being from work Work-life integration Basic needs for thriving Job design & experience of work Health, physical, and mental well-being from work	27

**6**	Neidlinger et al. (2024) Short version	A single factor of thriving	8


The table shows that the factors of thriving fail to converge, with the number ranging from 1 to 7. The factors are also different for those who attempted to conceptualize it. For instance, Smith et al. (2023) identified WPT as a single-factor instrument with six items. A recent short-form scale by Neidlinger et al. (2024) had a single factor with eight items. Spreitzer et al. (2005) proposed WPT as a two-dimensional construct comprising vitality and learning. In line with this, Porath et al. (2012) and Na-Nan et al. (2020) identified thriving as vitality and learning. Porath et al. (2012) modeled WPT as a second-order factor, viewed subjective experience, and identified learning as a stable personality trait. Learning orientation examines the general desire to learn. They developed five items that reflect a momentary sense of learning. According to self-determination theory, vitality is conceptualized as the energy emanating from individual actions. They developed five items under vitality. However, the scale by Na-Nan et al. (2020) identified 12 items. The scale by Neidlinger et al. (2024) had six factors with 27 items. Peters et al. (2021) developed an 87-item, 6-factor scale. The factors of all the social scientists were different, with no convergence.

Thus, prior research shows that thriving is essential because it enhances performance, innovation, and employee well-being. It has been identified as a psychological state limited to vitality and learning, and the literature has remained conceptually and dimensionally fragmented. Most studies have been narrowly focused on individual predictors, treating them in isolation rather than as synergistic psychological resources, including variables such as resourcefulness, mindfulness, or resilience. Mindfulness enhances alertness, regulates emotions, and impacts thriving. Resilience serves as a buffer against burnout. However, its contribution to thriving is scarcely examined. Only a limited number of empirical studies have examined resourcefulness and its complementarity with self-regulatory resources that promote adaptive functioning and thriving. These extreme divergence in factors and items calls for reconceptualizing WPT. The present study addresses these gaps in the literature by adopting an integrative approach that considers mindfulness, resourcefulness, and resilience as mutually reinforcing components of WPT. By positioning these three components within an integrated framework, this study seeks to resolve inconsistencies and extend understanding of how several psychological resources converge to support sustained well-being and enhance performance.

### Reconceptualization

Workplace thriving is a macro-indicator that helps individuals develop in a positive direction. A synthesis of the available literature suggests that thriving is associated with high levels of well-being and may facilitate development (Ryan and Deci, 2001). Chang and Busser (2019) proposed that a series of specific work contexts create conditions that facilitate thriving. The reconceptualization is thus grounded in a comprehensive literature review and a robust methodology. As discussed in the methods section, this study adopted a pluralist approach to reconceptualization. The linguistic method (Berenskoetter, 2016; Guzzini, 2013; Ish-Shalom, 2021), the “ladder of abstraction” (Sartori, 1970), and constructive discourse were utilized to reconceptualize WPT. These approaches helped replace scholarly disagreements and foster constructive discourse about WBT, thereby reconsidering its current boundaries (Knott and Alejandro, 2024). It has also helped refine and redefine the construct, presenting a comprehensive framework.

The literature suggested that the combined effect of a few contextual, proximal, and resource factors promotes WPT. Contextual factors include a climate of trust and respect, information sharing, and the discretion to make decisions. In addition to these predictors, there are proximal factors, including “agentic work behaviors” such as task focus, exploration, and mindful relating. These predictors are proximal because individuals who act agentically experience instant vitality and learning. Other variables that enhance WPT include workplace resources—such as knowledge, positive meaning, and affect—and relational resources. Unlike contextual features, these characteristics are renewable because they are endogenously created through workplace social interactions. These factors are presented below:

1. **Contextual factors**: Contextual work factors involve external or situational elements that shape employees’ experiences and behaviors at work. The mindfulness context factor is a mental state characterized by focused awareness and presence, shaping how individuals perceive and respond to their work context (Alruwayti and Sulphey, 2024; Jiang et al., 2022; Shapiro and Carlson, 2017). It directly affects how individuals engage with their work environment (Jnaneswar and Sulphey, 2021) and enhances emotional regulation, creativity, stress management, and task attention, impacting workplace interactions, decision-making, and overall well-being (Byrne and Thatchenkery, 2019; Zoghbi-Manrique-de-Lara et al., 2019). Hence, although mindfulness originates internally, it influences an individual’s approach to the external work context, making it a relevant contextual work factor that fosters a positive work environment and thereby enhances resilience and performance.
2. **Proximal factors**: Proximal work factors refer to personal characteristics or capabilities that directly impact individual behavior and performance. Resourcefulness is a proximal factor that directly influences an individual’s immediate approach to challenges and problem-solving. It involves enduring generic competencies that enable adaptive responses to organizational demands (Scheidgen et al., 2025). Resourcefulness involves using resources creatively to solve organizational problems (Rubaca and Khan, 2022) and is closely linked to an individual’s current decisions and actions. Individuals with higher levels of resourcefulness tend to experience greater empowerment in their work environments (Kanungo and Menon, 2004). Resourcefulness, as a proximal factor, enhances resilience, innovation, and success in routine tasks, equipping individuals to address immediate challenges effectively.
3. **Resource factors**: These are personal or environmental assets that support individuals in achieving their goals or maintaining well-being. Resilience is an internal resource that helps to cope with and adapt to stress, adversity, and challenges (Masten and Reed, 2002; Treves et al., 2025). Resilience enables individuals to recover from stress, demonstrating dynamic psychological adaptation and essential coping traits (Masten, 2001). It is a valuable resource that provides a psychological buffer, helping individuals recover from setbacks, persist through difficulties, and maintain productivity (Sulphey, 2020). It is a resource factor that strengthens one’s ability to handle pressures and contributes to sustained performance, well-being, and adaptability.

Based on the literature, the authors derive **mindfulness, resourcefulness**, and **resilience** as contextual, proximal, and resource factors, respectively. The dimensions are presented in Table 2 and the following sections.

**Table 2:** Reconceptualized WPT.


CONCEPT	DEFINITION	DIMENSIONS	DEFINITION

**Workplace thriving**	A state of positive engagement and well-being is derived when employees are mindful, resourceful, and resilient, enabling them to navigate organizational challenges and achieve effectiveness.	**Mindfulness**	Attention and non-judgmental awareness enable individuals to recognize their internal experiences and, in no way, automatically react or become overwhelmed.

		**Resourcefulness**	“A repertoire of cognitive-behavioral self-control skills acquired by individuals throughout their lives to cope effectively with stressful life events and to execute self-control behaviors successfully.”

		**Resilience**	The “capacity and dynamic process of adaptively overcoming stress and adversity while maintaining normal psychological and physical functioning.”


Mindfulness is derived from Jnaneswar and Sulphey (2021) and Shapiro and Carlson (2017). It is a meta-cognitive variable that is strongly associated with meta-emotion. Meta-emotion is being aware, responsive, and regulating emotional states. It strongly connects to meta-emotion, as it empowers individuals to be more aware of their feelings and emotions and respond to and regulate them. Mindfulness involves attention and non-judgmental awareness, enabling individuals to recognize their internal experiences and cultivate a growth mindset without automatically reacting or becoming overwhelmed (Holloway, 2025; Kabat-Zinn, 1990), and to bring about the required behavioral change (Schuman-Olivier et al., 2024). It is achieved when an individual is alert and accepts thoughts and emotions arising from focused attention (Treves et al., 2025). Hence, as a meta-emotive variable, mindfulness reflects why individuals experience emotions, how they regulate or alter them, and their awareness of emotional responses. There exist multiple standardized scales to measure mindfulness. A few of them include Baer et al. (2006), Brown and Ryan (2003), He et al. (2023), Khoury et al. (2022), and Zheng et al. (2022).

Social scientists define resourcefulness in multiple ways (Li et al., 2018). Resourcefulness involves utilizing resources creatively to address organizational challenges. Closely linked to current decisions and actions, this variable is derived from the work of Rubaca and Khan (2022). It involves how “an individual responds to a situation in his or her life that causes the individual stress” (Zauszniewski et al., 2005). In the organizational context, resourcefulness is defined as a cluster of generic competencies that enable adaptive responses to the demands of managerial roles (Sahin et al., 2015). Rosenbaum (1990) defined it as “a repertoire of cognitive-behavioral self-control skills acquired by individuals throughout their lives to cope effectively with stressful life events and to execute self-control behaviors successfully.” It is a critical component of “meta-cognitive competence,” which involves finding timely and clever ways to deal with situations that require creativity, adaptability, and problem-solving. Resourcefulness requires cognitive awareness, which is the knowledge of one’s relevant mental resources and problem-solving strategies. In short, being resourceful involves effectively using one’s cognitive resources to solve issues. It is a complex interplay of various thought processes, including reasoning, appraisal, strategy realignment, and adaptation to situations, to achieve desired goals. Resourcefulness also helps individuals regulate and direct their behaviors to endure adverse environments and sustain various coping behaviors (Rosenbaum, 1990). A few scales used to measure resourcefulness include those by Harris et al. (2006), Kanungo and Menon (2004), Rossetti and Zlomke (2021), and Zauszniewski et al. (2006).

Resilience is derived from the work of Haight et al. (2002). It involves the “capacity and dynamic process of adaptively overcoming stress and adversity while maintaining normal psychological and physical functioning” (Russo et al., 2012). When faced with various psychosocial and organizational challenges and demands, resilient individuals better understand their potential and abilities to navigate and confront demanding situations, thereby learning and responding more effectively. Such individuals also exhibit long-term orientation and risk propensity (Sulphey, 2020) and retain robust confidence, abilities, and mental well-being (de la Fuente et al., 2017). Hence, resilience is identified as a meta-motivational variable (de la Fuente and Amate, 2019). Meta-cognition involves knowledge about knowledge and the control of cognitive processes (Pintrich, 2002; Schneider, 2008). It encompasses knowledge of personal characteristics and strategies that facilitate the addressing and accomplishment of tasks (de la Fuente et al., 2017). Learning is a process that requires significant motivation. On the positive side, learning ensures meeting all adaptive demands and responses. The meta-motivation framework examines whether individuals recognize distinct types of motivation that facilitate achieving goals in response to situational requirements (Miele et al., 2020). This framework proposes performance trade-offs that can be relatively beneficial for some tasks but detrimental for others. It also diminishes the exacerbation of emotional distress, helplessness, and apathy (Alvarez-Ramírez and Cáceres, 2010). Resilient individuals also possess energy and vitality, and can effectively deal with adversity (Cohn et al., 2009; Porath et al., 2012; Na-Nan et al., 2020; Sulphey, 2020; Sulphey and Jasim, 2025). Energetic and vital individuals perform their tasks effectively. The most common scale for measuring resilience is the Connor-Davidson Resilience Scale (2003). A few other scales include those of Ruparel et al. (2022) and Smith et al. (2008).

Thriving individuals will be able to meta-cognize (manage their thinking), be meta-emotive (aware of their emotions), and meta-motivate (remain motivated or realign their motivation levels based on their goals and objectives). Integrating these three processes forms a holistic and resilient self-regulation system for individuals. This holistic self-regulation system enables individuals to monitor, regulate, and optimize their thought processes, emotions, and motivations, thereby fostering resilience and achieving their goals more effectively. Thus, this integrated system is crucial for adaptive functioning and resilience in complex and dynamic environments. Furthermore, this integration enables better and more effective performance. Musil et al. (2021) identified that these three variables are theoretically discrete but related constructs essential for enhancing well-being.

**Figure 1:**
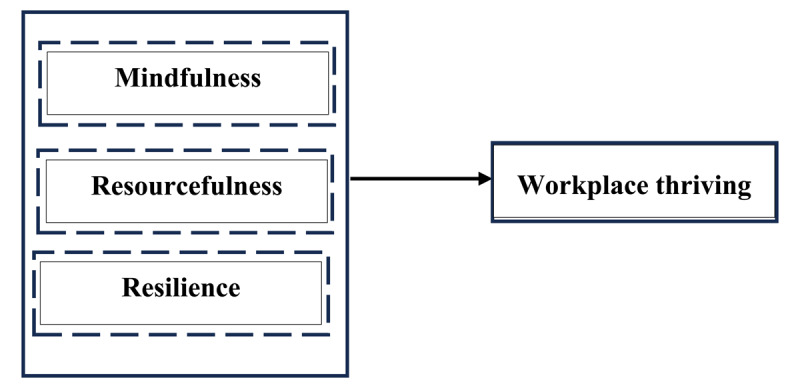
Reconceptualized Workplace Thriving.

Based on these, WPT is construed as a higher-order construct involving mindfulness, resourcefulness, and resilience (Figure 1), which can be positively associated with positive relational attributes, such as vitality, good interpersonal relationships, resources, social interactions, and high-quality connections among subordinates, peers, supervisors, and other stakeholders. Hence, a new definition of WPT is proposed. This study defines WPT as **“*a state of positive engagement and well-being derived when employees are mindful, resourceful, and resilient, enabling them to navigate organizational challenges and achieve effectiveness***.” This reconceptualization, consistent with prior social science research, assumes that WPT can shape an organization’s social and relational atmosphere, influencing citizenship behavior, engagement, overall organizational culture, and success. It is an adaptive function that enables individuals to navigate and adapt their work contexts to foster positive development. When there is WPT, employees exhibit agentic behaviors (Wrzesniewski and Dutton, 2001), which enable them to continually focus on tasks, enhance exploratory behavior, and mindfully build additional resources, further fueling, sustaining, and reinforcing WPT.

## Discussion

Thriving is a multifaceted construct with distinct meanings for different individuals. Hence, it is challenging to integrate existing literature and establish a clear operational definition that accurately reflects the construct. It also enjoys contextual variance, and scholars have adopted multiple conceptualizations based on the investigated domain (Brown et al., 2017). For instance, scholars examining thriving in developmental domains have conceptualized it as a developmental and growth-oriented process (Benson and Scales, 2009; Bundick et al., 2010). Su et al. (2014) opine that successful development is obtained through holistic functioning. Saakvitne et al. (1998) conceptualized WPT as a continuum, ranging from thriving to not thriving. Carver (1998) conceived thriving as a positive psychological growth experience that “energizes and enlivens.” In organizations, thriving is characterized by a sense of accomplishment, prosperity, success, and wealth (Bakker et al., 2010; Jackson et al., 2011; Sarkar and Fletcher, 2014). These domain-specific conceptualizations have resulted in multiple definitions (Sarkar and Fletcher, 2014). However, there are debates over whether thriving is a state, a process, or a combination of both, and whether it is domain-specific or requires a generalized realization (Brown et al., 2017; Sarkar and Fletcher, 2014). These divergent conceptualizations and definitions pose challenges for scholars, as conceptual clarity and convergence provide directions and boundaries for scientific and empirical examination (Kaplan, 1964). To address these issues, a comprehensive and robust definition applicable across diverse populations and domains is necessary.

Su et al. (2014, p. 272) stated that thriving “is not only marked by feelings of happiness, or a sense of accomplishment, or having supportive and rewarding relationships, but is a collection of all these aspects.” This conceptualization is broader than earlier definitions proposed by researchers like Park (1998), who focused on scenarios; Spreitzer et al. (2005), who focused on contexts; and Benson and Scales (2009), who focused on temporal restrictiveness. Thus, recent conceptualizations identify thriving as a global and comprehensive construct in which individuals strive to thrive across broad areas of their lives. Coe-Nesbitt et al. (2021) identified thriving as a complex, multi-dimensional construct involving multiple overarching themes. Others, for instance, Peters et al. (2023), identified WPT as a holistic concept of work-related well-being encompassing positive mental, physical, and social functioning, enabling maximum potential. They opined that WPT would prevent exhaustion and provide physical and psychological safety and growth. All this evidence points towards non-convergence about the concept of WPT. Hence, based on an extensive literature review, this study reconceptualized WPT as “a state of positive engagement and well-being derived when employees are mindful, resourceful, and resilient, enabling them to navigate organizational challenges and achieve effectiveness.” The factors identified to constitute WPT include mindfulness, resourcefulness, and resilience.

Mindfulness is “paying attention in a particular way: on purpose, in the present moment, and nonjudgmentally” (Kabat-Zinn, 1994, p. 4). It is “a state of alertness and lively awareness” achieved by active information processing, characterized by cognitive differentiation (Langer, 1989). Resourcefulness is “a set of generic competencies that enables adaptive responses to the demands of the managerial role.” Kanungo and Menon (2004). The resourcefulness framework comprises three generic competencies: affective, intellectual, and action-oriented (Kanungo and Misra, 1992). Affective competence encompasses emotional control that effectively manages intense emotions. Intellectual competence involves problem-solving and self-reflection. Action-oriented competence encompasses task-related proficiencies, such as persistence and attention to the timeline, as well as people-oriented skills, such as interpersonal acuity and compassion. Resilience is “effective coping and adaptation, although faced with loss, hardship, or adversity” (Tugade and Fredrickson, 2004). Individuals with high levels of resilience use positive emotions to “bounce back” from adverse experiences (Carle and Chassin, 2004).

### Future research agenda

This study develops a renewed understanding of WPT and its dynamics by proposing a reconceptualized, comprehensive framework that showcases its intricate interplay at the workplace. This framework explains the concept in the backdrop of contextual, proximal, and resource factors, deriving from mindfulness, resourcefulness, and resilience, respectively. The lack of these could impair WPT and manifest in counterproductive workplace outcomes. Thus, this work contributes to the literature by integrating the interdimensional connexions and agentic behaviors inherent in the WPT concept into the theory of self-adaptation (Tsui and Ashford 1994). There exist multiple opportunities for qualitative and quantitative examination of WPT. Researchers can make further distinctive and valuable contributions by empirically examining it. Detailed qualitative studies could also identify the facilitators and constraints that facilitate WPT. Further studies could be undertaken to develop a comprehensive WPT scale.

This study has multiple implications. The reconceptualized WPT represents a paradigm shift from how this concept was outlined, understood, and applied. This study provided a fresh perspective, revisiting the concept’s foundations, challenging earlier assumptions, and proposing a new definition. This reconceptualization has implications across various theoretical, practical, and academic domains. Theoretically, since the study employed an innovative, pluralistic methodological approach, it enhanced clarity of the concept. The reconceptualized WPT can help scholars, practitioners, social scientists, and management experts develop a practical, effective, and nuanced approach to address complex organizational issues. Although applying the revised definition and model may pose challenges, this paper provides valuable insights into WPT by highlighting its dynamic nature. Additionally, future research could examine how the three components of WPT (mindfulness, resourcefulness, and resilience) temporally interact to influence other workplace resources. Studies could also explore various behavioural moderating factors, including the work environments that could impact thriving. Cross-cultural studies could help identify how cultural values and norms shape the three components in promoting WPT. Researchers could also attempt to develop and validate a tool to measure the construct. Empirical studies could also be conducted to determine whether the synergy between the various components varies across industries or cultural contexts.

## Conclusion

A thorough review of the literature on WPT revealed a general lack of clarity about the concept, characterized by definitional, dimensional, and conceptual divergence. Hence, this work has attempted to introduce an element of convergence, focusing on contextual, proximal, and resource factors. Based on these factors, the three different dimensions derived include mindfulness, resourcefulness, and resilience. This reconceptualization presents an initial argument for understanding WPT, which helps individuals navigate their organizational lives more effectively and efficiently. In conjunction with existing literature on thriving, this reconceptualization framework explains WPT across organizations. This framework, describing the propositions and three factors that shape WPT, offers researchers a specific agenda for further empirical examination. This research has also emphasized implications for thriving in organizations, and researchers could employ this framework to sustain and reinforce WPT.
